# Efficacy of different suturing techniques on gingival grafts: A scoping review

**DOI:** 10.34172/japid.025.3805

**Published:** 2025-09-25

**Authors:** Mina Shekarian, Shiva Shekarian, Mahboobe Heydari, Zohreh Afshari, Romina Meshkinnejad

**Affiliations:** ^1^Dental Research Center, Dental Research Institute, School of Dentistry, Isfahan University of Medical Sciences, Isfahan, Iran; ^2^School of Dentistry, Shahid Beheshti University of Medical Sciences, Tehran, Iran; ^3^Dental Implants Research Center, Dental Research Institute, School of Dentistry, Isfahan University of Medical Sciences, Isfahan, Iran; ^4^School of Management and Medical Informatics, Isfahan University of Medical Sciences, Isfahan, Iran; ^5^Department of Periodontics, Dental Implants Research Center, Dental Research Institute, School of Dentistry, Isfahan University of Medical Sciences, Isfahan, Iran; ^6^Dental Students’ Research Committee, Department of Periodontics, School of Dentistry, Isfahan University of Medical Sciences, Isfahan, Iran

**Keywords:** Connective tissue, Gingival grafting, Gingival recession, Sutures, Wound healing

## Abstract

**Background.:**

This review evaluated the efficacy of various suturing techniques in gingival graft stabilization to optimize clinical outcomes and minimize the need for revision surgeries.

**Methods.:**

This scoping review was conducted across Scopus, PubMed, Cochrane, Web of Science, and ProQuest (through April 2025) using PICO criteria: Population (gingival grafts around teeth), Intervention (different suturing techniques), Comparison (efficacy of various suturing techniques in gingival graft stabilization), and Outcomes (keratinized tissue width [KTW], keratinized tissue height [KTH], and root coverage [RC]). From 838 initial records, 73 studies met the inclusion criteria after dual-reviewer screening with arbitration by a third reviewer. Study quality was assessed using the Joanna Briggs Institute tools.

**Results.:**

For free gingival grafts (FGGs), primary stabilization methods included interrupted sutures (with/without periosteal fixation), sling sutures, and cyanoacrylate. Connective tissue grafts (CTGs) predominantly use sling sutures, often combined with cross-mattress or interrupted sutures, vertical/double-cross mattress techniques, or continuous sutures with coronally advanced/tunnel flaps. While 72% of FGG studies (23/32) reported significant KTW improvement with interrupted sutures (a mean gain of 2.1±0.8 mm), CTG studies demonstrated 96% RC success (43/45) with sling-based techniques. However, outcomes showed substantial heterogeneity due to variability in the Miller classification (33/67 studies focused on Class I only) and inconsistent reporting of suture material (only 5/67 specified size/type).

**Conclusion.:**

No single suturing technique demonstrated clear superiority in graft stabilization, likely due to study heterogeneity. While sling/mattress combinations showed optimal RC for CTGs and interrupted sutures/cyanoacrylate performed well for FGGs, standardized RCTs controlling for confounding variables are required to establish definitive protocols.

## Introduction

 Gingival grafting is a frequently performed procedure in periodontal surgery to repair lost gingival tissue. Gingival grafts are classified into three main categories: autografts, xenografts, and allografts, each with its subgroups. The two most used types of gingival autografts are free gingival grafts (FGGs) and connective tissue grafts (CTGs).^1^

 FGG is ideal for areas with low aesthetic demands or when a significant volume of keratinized tissue is required.^2^ Additionally, FGG has a low risk of complications and can be easily harvested. However, some drawbacks of FGG include donor site morbidity, limited blood supply, and difficulty in achieving an aesthetically pleasing outcome due to poor color matching.^3-6^

 CTG is suitable for areas with high aesthetic demands or where precise contouring is needed due to its thinner tissue biotype.^2^ Its applications include increasing the gingival width,^7^ root coverage (RC),^8,9^ alveolar ridge augmentation,^10,11^ addressing peri-implant tissue abnormalities,^12^ and even coverage of fenestration.^13^ Additionally, CTG has a better blood supply, leading to faster healing and improved tissue integration compared to FGG.^2^

 Suturing technique plays a critical role in gingival graft success by ensuring tissue stabilization. The main groups of suturing methods include periosteal suture, interrupted suture, sling suture, mattress suture, cross-suture, and continuous suture.

 This review examines the efficacy of various types of sutures for gingival graft stabilization, aiming to enhance clinical outcomes and minimize the need for revision surgeries.

## Methods

 A systematic scoping review of clinical trials was developed, considering the PRISMA (Preferred Reporting Items for Systematic Reviews and Meta-analyses) extension for scoping reviews.^14^ The protocol of this study was based on the framework proposed by Peters et al^15^ according to the Joanna Briggs Institute. The protocol was registered in Open Science with the code number 10.17605/OSF.IO/4YR9F. In addition, this project was approved by the Ethics Committee of Isfahan University of Medical Sciences (IR.MUI.DHMT.REC.1403.133).

 This scoping review aimed to respond to the following focused question. In patients undergoing soft tissue grafts, (a) what methods are used for graft stabilization? (b) What is the efficacy of different suturing techniques on graft success?

 The PICO for the present review was as follows:

- Population (gingival grafts around teeth)
- Intervention (different suturing techniques)
- Comparison (efficacy of various suturing techniques in gingival graft stabilization)
- Outcomes (keratinized tissue width [KTW], keratinized tissue height [KTH], RC).

###  Selection criteria

 We included randomized and non-randomized controlled clinical trials, cohort studies, case reports, and case series that considered at least one type of soft tissue grafting techniques and mentioned the suturing method. Only studies written in English were included. Exclusion criteria included animal studies, in vitro studies, finite element analysis (FEA) studies, letters to the editor, reviews, and publications about soft tissue grafts around dental implants.

###  Search strategy

 An electronic search of articles in English, with no time restrictions, was conducted in Scopus, PubMed, Cochrane, Web of Science, and ProQuest, up to April 2025. The following search model was accomplished using Boolean operators (“Gingival graft*” OR FGG OR “Free gingival graft” OR CTG OR “Connective Tissue graft” OR “Phenotype Modification gingival”) AND Suture* in TITLE/SUBJECT/ABSTRACT based on the particular search strategy of each database (Table 1). A manual search (2000–2025) was performed in the *Journal of Dental Research*, *Journal of Clinical Periodontology*, *Journal of Periodontology*, *Clinical Oral Implant Research*, *Clinical Implant Dentistry and Related Research*, *International Journal of Oral and Maxillofacial Implants*, and *Journal of Oral and Maxillofacial Surgery*. Additionally, the reference section of the included studies (cross-referencing) was screened for potential further studies.

###  Screening

 After removing duplicates, both automatically (by using Mendeley reference manager software (Version 2.110.2) and manually, the titles and abstracts of the search results were initially screened by two independent authors (M.SH. and SH.SH.). Publications were included for full-text evaluation if the study met the inclusion criteria during the initial analysis or for studies with insufficient information from the title and abstract. Disagreements between the authors were resolved by discussion. In the event of disagreement, the opinion of a third reviewer (Z.A.) was sought. Following full-text assessment, studies were either selected for inclusion or rejected. In papers that included inadequate or limited information about suturing technique, the corresponding authors were contacted via email for clarification or to request missing data, and a reminder was sent twice later.

###  Data extraction

 The following data were extracted from the included studies for further investigations, which are summarized in Table 2: First author, country, study design, number of patients/teeth, site of grafting, grafting technique, type of suture, reported outcomes (KTW, gingival tissue thickness [GTT], clinical attachment level [CAL], KTH, probing depth [PD], attached gingiva [AG], RC, recession depth [RD] and other relative outcomes).

###  Outcome measures

 The primaryoutcomes were GTT and KTW. The secondaryoutcomes included all other reported measures: CAL, PD, RC, KTH, and vestibular depth (VD).

## Results

**Figure 1:**
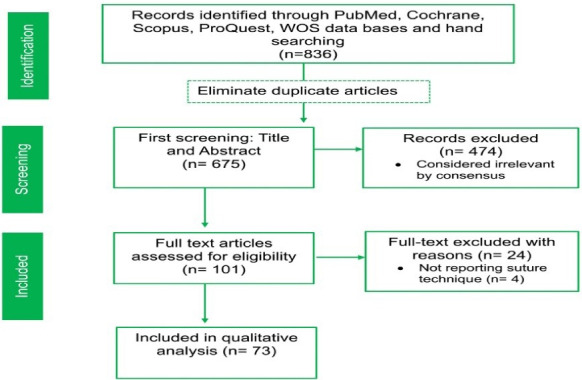
PRISMA flowchart for selecting articles

 The PRISMA flow diagram illustrates the study selection process at various stages, as depicted in Figure 1.

 Some studies meeting our subject criteria were excluded because their suturing techniques were unclear. Although we contacted the corresponding authors for clarification via email, no responses were received, necessitating their exclusion.^71,88-90^

**Figure 2:**
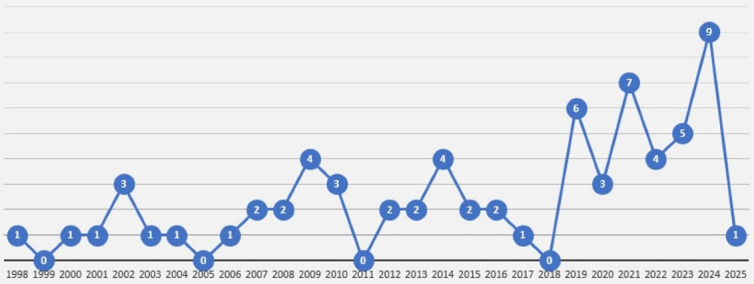
The frequency of the included articles from 1998 to April 2025

**Figure 3:**
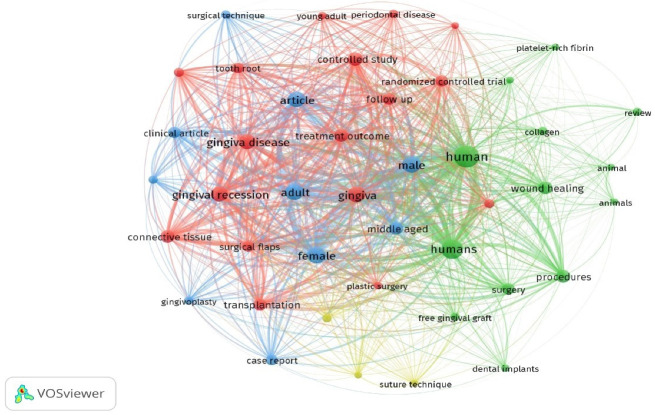
The frequency and keyword co-occurrence network generated using VOSviewer (version 1.6.20) based on titles and abstracts of the included studies. The data were extracted from the titles and abstracts of all the included articles. The font size and linkage thickness reflect the frequency of each keyword and the strength of co-occurrence between terms, respectively. Larger nodes and bolder text indicate higher frequency and stronger relational clustering within the dataset

Figure 2 presents the frequencies of the included articles from 1998 to 2025. Figure 3 presents the frequencies and relationships between the keywords of the articles.

###  Free gingival graft 

**Figure 4:**
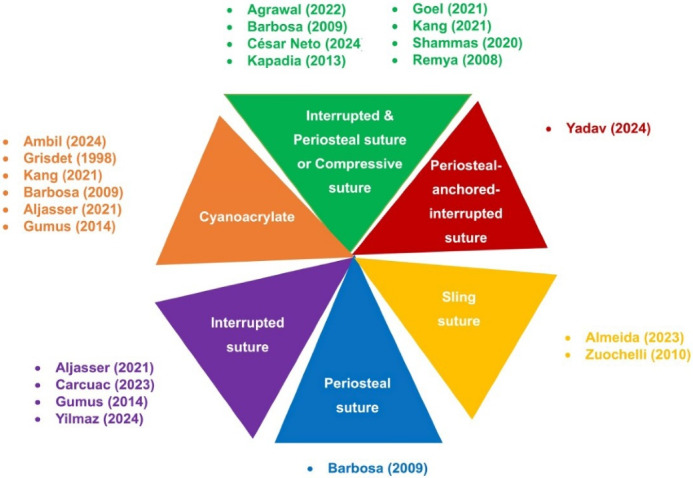
Various techniques to stabilize the FGG

 Research on the use of the FGG technique for soft tissue augmentation includes three case report studies,^39^ seventeen randomized controlled trials (RCTs),^29,33,42,45,56,57,61-63,67,68,70,82,86,87,91,92^one technical note study,^48^ and two case series.^4,9^ These studies employed various techniques to stabilize the FGG, such as interrupted sutures (direct loop), sling sutures, modified sling suture, and adhesive materials like cyanoacrylate (Figure 4). Below, the outcomes are organized by clinical parameters, along with the studies that measured them.

 Studies that measured PD reported mixed results: while Agrawal et al,^67^ Goel et al,^61^ and Barbosa et al^29^ found no significant change in PD, Menceva et al^92^ and Chelearescu et al^57^ observed a reduction in PD. Conversely, Carnio et al^45^ and Remya et al^4^ reported a significant increase in PD.

 KTW was a commonly measured parameter, with most studies reporting an increase.^39,45,57,61,62,67,82,86,87,92^ However, some studies found no significant changes in KTW.^29,61^

 CAL was evaluated in several studies, with varying outcomes: Goel et al,^61^ Kang et al,^62^ and Remya et al^4^ reported improvements in CAL, whereas Agrawal et al,^67^ Yilmaz et al,^86^ and Barbosa et al^29^ found no significant changes.

 Gingival recession (GR) was another key parameter, with most studies reporting a reduction.^57,61,62,67,92^ However, some studies found no significant difference between the groups.^29,61^

 RC and complete root coverage (CRC) were evaluated in a subset of studies: Cortellini et al,^37^ Chelearescu et al,^57^ and Remya et al^4^ achieved RC, while Chelearescu et al^57^ and Shakiliyeva et al^87^also reported achieving CRC.

 GTT was measured in a few studies, with Goel et al^61^ and César Neto et al^82^ reporting an increase in GTT.

 Some studies focused on postoperative outcomes, such as pain and shrinkage: Alhourani et al^68^ reported that pain persisted for up to 4 days, with complete healing within 2 months, and noted that the cyanoacrylate group experienced less pain at 6 hours but no significant difference in long-term outcomes. Additionally, the same study observed significantly less shrinkage in the cyanoacrylate group after 3 months.

**Table 1:** Specific search strategy for each database

**Database**	**Search Strategy**
Web of Science	TS = ((“Gingival graft*” OR FGG OR “Free gingival graft” OR CTG OR “Connective Tissue graft” OR “Phenotype Modification gingival”) AND Sutur*)
PubMed	(“Gingival graft*”[Title/Abstract] OR FGG[Title/Abstract] OR “Free gingival graft”[Title/Abstract] OR CTG[Title/Abstract] OR “Connective Tissue graft”[Title/Abstract] OR “Phenotype Modification gingival”[Title/Abstract]) AND Sutur*[Title/Abstract]
Scopus	TITLE-ABS-KEY ((“Gingival graft*” OR FGG OR “Free gingival graft” OR CTG OR “Connective Tissue graft” OR “Phenotype Modification gingival”) AND Sutur*)
Embase	(‘Gingival graft*’:ti,ab,kw OR FGG:ti,ab,kw OR ‘Free gingival graft’:ti,ab,kw OR CTG:ti,ab,kw OR ‘Connective Tissue graft’:ti,ab,kw OR ‘Phenotype Modification gingival’:ti,ab,kw) AND (Sutur*:ti,ab,kw)
ProQuest	((“Gingival graft*” OR FGG OR “Free gingival graft” OR CTG OR “Connective Tissue graft” OR “Phenotype Modification gingival”) AND Sutur*)

**Table 2:** Detailed characteristics of included articles

**Author (Year)**	**Study design**	**Recession site**	**N. Patients /sites-teeth**	**Graft technique**	**Outcome measures**
Nelson (1987)^16^	Case series	Not mention	14 P	CTG + Double pedicle	CRC, RC
Grisdale (1998)^17^	Case report	Case 1: Mandibular incisors Case 2: Biopsy site	2 P	Case 1: FGG Case 2: FGG	RC
Rosetti et al (2000)^18^	RCT	Miller Class I or II gingival recession (upper canine or premolars)	24 S	Group 1: CTG + CAF Group 2: GTR	KTW, PD, RC, PI, GI
Cordioli et al (2001)^19^	RCT	Miller Class I or II gingival recession	21 P /62 S	Group 1: CTG + Envelope Group 2: CTG + CAF	KTW, RC
Tal et al (2002)^20^	RCT	Class I or II Miller classification ≥ 4 mm in the apicocoronal dimension	14 P	Group 1: ADM + CAF Group 2: CTG + CAF	RD, RW, KTW, PD, CAL
Carnio et al (2002)^21^	Case series	Miller’s Class II and III gingival recession	4 T	CTG + EMD + CAF	PD, CAL, KTW
Paolantonio (2002)^22^	RCT	Miller Class I or II gingival recession	45 S	CTG + CAF	PD, CAL, KTW, GTT
McGuire & Nunn (2003)^23^	RCT	Miller’s Class II gingival recession	17 P	Group 1: EMD + CAF Group 2: CTG + CAF	RD, RW, KTW, PD, CAL
Cheung & Griffin (2004)^24^	RCT	Miller’s Class I or II gingival recession	15 P / 54 T	Group 1: Platelet concentration + CAF Group 2: SCTG + CAF	VRD, RW, KTW, PD, CAL
Carvalho et al (2006)^25^	Case series	Class I or II adjacent multiple gingival recession	10 P / 29 S	CTG + MCAF	PD, CAL, KTW, RD
Dembowska & Drozdzik (2007)^26^	Case series	Miller’s Class I or II gingival recession	18 P 48 S	Group 1: CTG + TUN Group 2: CTG + TUN	PI, RW, KTW, PD, RD
Felipe et al (2007)^27^	RCT	Bilateral Miller Cl I and II gingival recession	15 P	Group 1: ADM + CAF Group 2: ADM + CAF without releasing	PD, CAL, GR, KTW, GTT
Remya et al (2008)^4^	Case series	Early class III gingival recession	10 P	FGG	PD, CAL, RW, RD
Han et al (2008)^28^	RCT	Miller Class I and II gingival recessions	20 P	Group 1: exposed CTG Group 2: CTG + CAF	RD, PD, CAL, PI, KTW
Barbosa et al (2009)^29^	RCT	Buccal sites of mandibular incisors and Miller’s class I or II recessions	24 P	Group 1: cyanoacrylate Group 2: FGG	PD, GR, CAL
Cortellini et al (2009)^30^	RCT	Single Miller Class I and II buccal gingival recessions	85 P	Group 1: CTG + CAF Group 2: CAF	RD, PD, KTW, CAL
Bittencourt et al (2009)^31^	RCT	bilateral Miller Class I gingival recessions (4 mm) in maxillary canines or premolars	17 P	Group 1: CTG + CAF Group 2: Semilunar Coronally Positioned Flap	RD, RW, PD, CAL, GTT, KTW
Byun et al (2009)^32^	RCT	Class I or II gingival recessions ‡2 mm on anterior teeth and premolars	20 P	Group 1: SCTG + CAF Group 2: SCTG with Epithelial collar + CAF	PD, REC, CAL, RW, KTW, PI, GI
Zucchelli et al (2010)^33^	RCT	single Miller’s Class I and II RED	50 P	Group 1: dFGG + CAF Group 2: CTG + CAF	RD, CAL, KTH, GTT
Aroca et al (2010)^34^	RCT	3 adjacent Class III gingival recessions	20P / 139 S	Group 1: CTG + MTUN Group 2: CTG + EMD + MTUN	PD, REC, CAL, KTW, RW, GI, PI
Pini-Prato et al (2010)^35^	Group 2 clinical trial	multiple recessions on both sides	13 P	Group 1: CTG + CAF Group 2: CAF	RD, PD, CAL
Cardaropoli et al (2012)^36^	RCT	single Miller’s Class I or II REC	18 P	Group 1: CTG + CAF Group 2: CM + CAF	REC, CAL, PD GTT, KTW
Cortellini et al (2012)^37^	Case series	12 single + 16 multiple recessions at lower incisors	19 p / 28 S	partially epithelialized FGG	KTW, RD
Aroca et al (2013)^38^	RCT	Multiple adjacent Miller class I and II gingival recession	22 P	Group 1: CM + MTUN Group 2: CTG + MTUN	RD, RW, CAL, PPD, KTW, GTT
Kapadia et al (2013)^39^	Case report	Labial aspects of mandibular central incisors	1 P	FGG	Attached gingival gaining
Moka et al (2014)^40^	RCT	Miller’s class I gingival recession defects in maxillary teeth.	20 P	Group 1: CAF Group 2: semilunar coronally repositioned flap	KTW, RD, PD, CAL
Zuhr et al (2021)^41^	RCT	Miller class I or II recessions for	24 P / 47 S	Group 1: CTG + TUN Group 2: EMD + CAF	PI, GI, PD, RD, KTW
Gümüş & Buduneli (2014)^42^	RCT	one or two lower anterior teeth, Miller Class III–IV recession	45 P	Group 1: FGG Group 2: FGG Group 3: FGG	KTW, shrinkage
Yaman et al (2015)^43^	Case series	One or multiple adjacent Miller Class III gingival recessions	9 P	CTG + MTUN	RC, KTW
Uraz et al (2015)^44^	RCT	Miller Class I and/or Class II GR in mandible or maxilla	20 P	Group 1: CAF + expanded mesh CTG Group 2: CAF + PRF	RC, RW, CAL, and KTW
Carnio et al (2015)^45^	RCT	Not mention	Group 1: 42 T Group 2: 35 T	Group 1: MARF Group 2: FGG	GR, PD, KTW
Cieślik-Wegemund et al (2016)^46^	RCT	Miller Class I and II gingival recession	28 P	Group 1: CM + TUN Group 2: CTG + TUN	CAL, PD, RD, CRC, KTW
Santoro et al (2016)^47^	Case report	Mandibular canine and a maxillary premolar	2 P	CAF + CTG + GTR	KTH, CAL, PD
Ku & Leem (2019)^48^	Case report	Vestibuloplasty on anterior mandible	1 P	FGG + Ti mesh	VD, KTW
Agusto et al (2019)^49^	Case report	Class II Miller buccal recession on #24	1 P	CTG + Gingival Pedicle With Split-Thickness Tunnel Technique	KTW, PD
Do (2019)^50^	Case report	Miller Cl I and II recession defects	1 P	CTG + VISTA	KTW, PD, RC
Damante et al (2019)^51^	RCT	Miller’s class I and II recession defects	17 P / 40 S	Group 1: CTG + CAF without root conditioning Group 2: CTG + CAF with root conditioning	RD, RC, KTW, GTT, PD, CAL
Baghele (2019)^52^	Case series	Not mention	6 P	CTG	Graft stabilization and survival
Rasperini et al (2019)^53^	Case series	Not mention	7 P	TUN	RC, GTT, and VD
Khuntia et al (2020)^54^	Case series	Miller’s Class I gingival recession	3 P	Case 1: PRF + CAF Case 2: CTG + CAF Case 3: CAF	RC
Bautista et al (2022)^55^	Case report	type I gingival recession on the vestibular surface of tooth 23	1 P	CTG + double papilla flap	RC
Shammas at al. (2020)^56^	RCT	in two quadrants of the mandible (premolar site)	10P / 20 S	Group 1: FGG Group 2: FGG	PD, KTW
Chelarescu et al (2020)^57^	RCT	gingival recession areas, class I and II Miller recession, with a recession depth of 2-5mm	12 P / 44 S	Group 1: FGG Group 2: CTG + CAF	RC, KTW, GR
Rakasevic et al (2020)^58^	RCT	Multiple adjacent Type 1 gingival recessions.	20 P	Group 1: CM + MTUN Group 2: CTG + MTUN	RC, KTW, GTT, RC
Salem et al (2020)^59^	RCT	Maxillary incisors, canines or premolars	40 P	Group 1: CTG + CAF Group 2: CTG + TUN/pouch	RC, CRC, GTT, KTW,
Cardoso et al (2021)^60^	cohort study	Miller Class I and II/ Cairo RT I) in maxillary or mandibular canines and pre-molars	60 P	CTG + CAF	RD, RW, KTW, GTT, RC
Goel et al (2021)^61^	RCT	Miller’s Class I and II gingival recession	48 S	Group 1: FGG Group 2: cyanoacrylate	RD, CAL, KTW
Kang et al (2021)^62^	RCT	Miller’s Class I and II	300 S	Group 1: FGG Group 2: cyanoacrylate	CAL, KTW
AlJasser et al (2021)^63^	RCT	lower anterior and premolar regions	22 P	Group 1: cyanoacrylate Group 2: FGG	KTW, GT, graft shrinkage
Agrawal et al (2021)^64^	Case report	Lingual aspect of mandibular lateral incisor	1 P	CTG + TUN	RC, KTW, shrinkage
Lee et al (2021)^65^	Case series	Miller Class I, II, and III gingival recession	17 P / 27 T	CTG + CM + modified TUN	RC
Rimbert & Barré (2021)^66^	Case report	Deep anterior mandibular recession	1 P	CTG + modified TUN	RC, attached gingiva
Agrawal et al (2022)^67^	Case series	Miller class I or II mandibular premolar region	17 P / 21 S	Modified FGG	RD, CAL, KTW, RC, PD
Alhourani et al (2022)^68^	RCT	gingival recession and the absence of the keratinized gingiva	12 P / 24 S	Group 1: cyanoacrylate Group 2: FGG	Graft shrinkage, postoperative pain
Tambe et al (2022)^69^	Case report	Miller Class I maxillary buccal gingival recession	3 cases	CTG + MTUN	RD, CRC, KTW
Carcuac et al (2023)^70^	RCT	mandibular incisors	30 P	Group 1: Modified FGG Group 2: FGG	RD, KTH, CRC
Alrmali et al (2023)^71^	RCT	Mandibular incisor area	40 P	Group 1: Modified gingival graft technique Group 2: FGG	KTW, GTT, RD, RW, GT, RC
Danskin et al (2023)^72^	Case report	Gingival recession on the lingual surfaces of teeth #22–27	1 P	CTG + TUN	RC, GTT, VD
Vilarrasa & Blasi (2023)^73^	Case report	lower incisors	1 P	CTG + Double laterally moved CAF	KTW, RC
Chang et al (2023)^74^	Case report	Not mention	1 P	bioceramic-based cement + CM + TUN	RC, CAL, PD
Kashani et al (2023)^75^	Case series	Cairo RT1	13 P	Molar or canine access CAF + CTG	CRC
Guimarães et al (2023)^76^	Case series	Multiple Miller’s class I, II and III recession	10 P/ 85 S	CTG + TUN	RD, RW, RC, CRC
Deepika and Thamaraiselvan (2023)^77^	Cohort study	Miller’s class I single or multiple tooth gingival recession	20 P	CTG + TUN	GI, PI, healing index, RC, RD
Santamaria et al (2025)^78^	Case report	RT1	1 P	CTG + CAF	CRC, PD, BOP, CAL, KTW, GTT, PI, GR
Yadav et al (2025)^79^	Case report	RT 1	2 P	labial gingival graft	RD, PD, KTW, attached gingiva, Postoperative pain, CRC
Rao et al (2024)^80^	RCT	Miller’s class I and II recession	20 S	CTG + CAF	RH, healing index, root coverage aesthetic score
Ambili et al (2024)^81^	Case report	Cairo’s RT2	1 P	FGG + laterally flipped periosteum	KTW, CRC
César Neto et al (2024)^82^	RCT	Mandibular anterior teeth	45 P	FGG	STT, STV, CA
Skierska et al (2024)^83^	RCT	Maxillary and mandibular anteriors	30 P	CTG + TUN	RC, KTW, GT, RES, MRC
Devkar et al (2024)^84^	RCT	Mandibular anteriors	40 S	CTG	GT, RC
Lin (2025)^85^	Case Series	Maxillary and mandibular anterior	3 P	CTG + Double-VISTA	RC, CAL gain, KT increase
Yilmaz et al (2024)^86^	RCT	Maxillary anterior	25 P	Group1: FGG Group 2: MCAT	KTW, GT, RC
Shakiliyeva et al (2025)^87^	RCT	Mandibular anterior region	25 P	Group 1: Gingival Unit graft Group 2: CTG	RC, KTW

FGG: free gingival graft; CTG: connective tissue graft; RC: root coverage; GT: gingival thickness; VD: vestibular depth; KTW: keratinized tissue width; CAG: clinical attached gingiva; KTH: keratinized tissue height; KTT: keratinized tissue thickness; PD: probing depth; RW: recession width; CAL: clinical attachment level; CRW: coronal recession width; ARW: apical recession width; PI: plaque index; GI: gingival index; RD: recession depth; STT: soft tissue thickness; GR: gingival recession; GRD: gingival recession depth; GRW: gingival recession width; BRW: buccal recession width; RH: recession height; RES; root coverage esthetic score; MARF: modified apically repositioned flap; GT: gingival thickness, STV: soft tissue volume; CA: creeping attachment.

###  Comparison of sutures vs. cyanoacrylate

 Several studies compared sutures with cyanoacrylate for FGG stabilization. While some found no significant differences between the groups,^29,61,62^ others reported greater RC in the cyanoacrylate group^62^ and less shrinkage with cyanoacrylate.^68^

###  Interrupted sutures for FGG stabilization

 Interrupted sutures are the most frequently used technique for stabilizing FGGs. Several studies have investigated the outcomes of this technique, including its impact on VD, keratinized tissue (KT), KTH, RC, and other clinical parameters.

###  Positive outcomes of interrupted sutures

 Interrupted sutures demonstrated several positive outcomes: a technical note study reported an increase in VD and KT,^48^ while Carcuac et al^70^ observed an increase in KTH and successful RC.

###  Comparison of FGG and modified FGG techniques

 Carcuac et al^70^ compared the traditional FGG technique with a modified FGG technique (using a connective tissue pedicle graft under the FGG). The modified FGG group showed a reduction in PD and RD, higher RC and KTH, and significantly lower postoperative morbidity.

 César Neto et al^82^ compared two FGG stabilization approaches: (1) a control group using interrupted sutures with periosteal suspensory sutures over the graft, and (2) a test group where the flap was sutured over the graft without periosteal sutures. Both techniques demonstrated significant increases in soft tissue thickness, with no statistically significant differences between the groups (*P* > 0.05).

###  Conflicting findings on interrupted sutures

 Despite the positive outcomes reported in many studies, some research has shown conflicting results. AlJasser et al^63^ found a slight decrease in KTW and a significant reduction in GTT during follow-up assessments. Their comparison of cyanoacrylate and suturing techniques showed no significant differences in mean KTW or mean FGG shrinkage; however, mean GTT increased significantly more in the suturing group.

###  Comparison of interrupted sutures, cyanoacrylate, and microsurgery

 Gümüş and Buduneli^42^ conducted a study comparing three FGG stabilization techniques: interrupted sutures, cyanoacrylate adhesive, and microsurgery. The interrupted suture group showed a decrease in PD, CAL, plaque index, and papilla bleeding index. In contrast, the cyanoacrylate group exhibited significantly lower graft shrinkage and recipient site pain compared to the other groups. The microsurgery group exhibited graft shrinkage, similar to the interrupted suture group.

###  Periosteal-anchored Interrupted Suture

 Yadav et al^79^ used a periosteal-anchored interrupted suture technique to stabilize labial gingival grafts for KT augmentation. Their study reported significant gain in keratinized tissue and CRC.

###  Sling Sutures

 Sling sutures are another technique used to secure FGGs.

###  Outcomes 

####  Keratinized tissue width 

 Almeida et al^91^ compared sling sutures (control group) with no sutures (test group). The control group showed a greater increase in KTW, though the difference was not statistically significant.

 Yilmaz et al^86^ demonstrated that FGGs stabilized with sling sutures yield superior long-term KTW gains (3.2 mm) compared to flap techniques.

###  Modified sling suture

 The sling suture with periosteal anchoring^87^ has demonstrated clinically significant improvements in graft survival, keratinized tissue gains, RC, and healing index compared to conventional sling sutures.^87^

###  Cyanoacrylate adhesive

 Cyanoacrylate has been explored as an alternative to sutures for FGG stabilization.

###  Outcomes

####  Postoperative pain and shrinkage

 Alhourani et al^68^ compared sutures with cyanoacrylate (Iceberg glue). The cyanoacrylate group experienced less postoperative pain at 6 hours and significantly less shrinkage after 3 months, though long-term outcomes were similar.

 Compared to sutures, the cyanoacrylate group experienced less postoperative pain at 6 hours and significantly less shrinkage after 3 months, though long-term outcomes were similar.^68^

 No significant differences were found in KTW or graft shrinkage between cyanoacrylate and sutures, but GTT increased significantly more in the suture group.^63^

 When compared to interrupted sutures and microsurgery, cyanoacrylate showed significantly lower graft shrinkage and recipient site pain.^42^

 Cyanoacrylate successfully achieved RC, with one study reporting CRC, increased VD, and reduced tooth morbidity.^17,81^

###  CTG

 Out of the studies that utilized the CTG technique for gingival reconstruction, twelve case report studies,^47,49,50,52,55,64,66,69,72,73,84,85^ six case series,^21,25,26,43,54^ one cohort study,^60^ twenty-five RCT studies,^18-20,22,23,28,30-34,36,38,40,41,44,46,51,57-59,65,74,83^ and a non-randomized controlled clinical trial^35^ were included. CTG secured with numerous suture methods and covered with various techniques, including tunnel flap, coronally advanced flap (CAF) and its modifications, lateral pedicle in addition to tunnel technique, semilunar coronally positioned flap, double papilla, and double pedicle in studies (Supplementary file 1, Table S1).

###  Tunnel flap technique

 While the tunnel and CAF techniques are commonly used in conjunction with CTG, a lack of uniformity is observed in securing the CTG or the entire graft complex in studies. Various suturing methods, such as sling, vertical mattress, double cross, horizontal mattress, V-reverse suture, and interrupted suture, have been utilized to secure the tunnel and its modification flaps.

###  Sling

 Most studies using the tunnel technique flap employed either sling sutures or a combination of sling sutures and another type of suture to secure the graft in place.^23,46,53,64,72,83^ All research that used only sling sutures found an improvement in RC.^46,53,72,76,77,83^ Furthermore, an increase in KTW,^46,83^ and GTT,^53^as well as a decrease in RD,^46^ were observed.

 Cieślik-Wegemund et al^46^compared the CTG and CM in their study and demonstrated that RC significantly increased in both groups. However, the mean RD showed a greater increase in the CM group; the mean RC and CRC increased significantly in the CTG group. The mean KTW increased almost equally in both groups.

###  Sling in addition to cross-mattress

 Agrawal et al^64^ used a sling suture in addition to a cross-mattress to secure the graft from lingual direction and reported that RC, KTW, and GT increased.

###  Vertical mattress suture

 Dembowska and Drozdzik^26^ reported an increase in KTW and RC similar to the Agrawal study; however, they used different suture methods (vertical mattress).

###  Double-cross suture

 Zuhr et al^41^ conducted a study comparing the tunnel technique with CTG and CAF with enamel matrix derivative (EMD) for RC. They used a double-crossed suture, along with interrupted sutures, to secure the grafts. The study reported that the tunnel technique with CTG had significantly better results. Both methods showed an increase in RC and a decrease in RD and CAL. However, there was a significant difference in RD and CAL between the tunnel flap with CTG and CAF with EMD. Additionally, the KTW increased in the tunnel flap with CTG but decreased in CAF with EMD.

###  Interrupted

 Salem et al^59^ conducted a study using interrupted sutures to secure the CTG in both the tunnel technique (TUN) and CAF techniques for treating GR. The study reported that TUN represented better long-term results. While TUN showed GT and KT were significantly better, there was no significant difference in RC between the two groups.

###  TUN modification techniques

####  Outcomes by clinical parameter

 Several studies have explored the use of TUN modification techniques for harvesting and stabilizing CTGs. Below, the findings are organized by clinical parameters and the studies that measured them.

 Studies evaluating RC and CRC demonstrated consistent improvements across various techniques. Using a coronally advanced modified tunnel technique with a horizontal mattress suture, improved RC and CAL were reported.^34^ Similarly, the MCAT technique combined with site-specific de-epithelialized gingival grafts (DGGs) and sling sutures demonstrated improved RC outcomes.^84^

 When comparing the CTG (control group) and CM (test group), the CTG group showed significantly higher CRC and mean RC.^38^ A similar comparison found improved RC in both groups, but the CTG group had significantly higher mean RC and CRC.^58^

 The use of bioceramics-based cement with CM also resulted in increased RC.^93^ Advanced techniques, such as TUN modification with interrupted sutures,^65^ a modified TUN technique with a V-reverse suture,^69^ and a double-crossed suture with the MCAT technique^43^ further enhanced RC and achieved CRC. Additionally, the VISTA approach, which incorporates a subperiosteal sling suture and horizontal mattress suture, resulted in increased RC.^50,83^

 When comparing double-VISTA (featuring dual vestibular incisions and subperiosteal tunneling) with CTG to conventional techniques, the double-VISTA group demonstrated significantly greater mean RC and CRC.^85^

 Studies evaluating KTW reported varied outcomes depending on the technique used. A coronally advanced modified tunnel technique showed no significant changes in KTW,^34^ while the CM group demonstrated an insignificant increase in KTW.^38^ Both CTG and CM groups exhibited improved KTW, with no significant differences between them.^58^ Significant increases in KTW were achieved using advanced techniques, such as a modified TUN technique with a V-reverse suture,^69^ a double-crossed suture with the MCAT technique,^43^ a sling suture with MCAT,^84^ a double-VISTA technique,^85^ and the VISTA approach, which incorporated advanced suturing methods.^50^

 Studies evaluating CAL demonstrated improvements across various techniques. Using a coronally advanced modified tunnel technique, improved CAL was reported.^34^ The MCAT technique also showed a significant reduction in CAL.^38^ When comparing CTG (control group) and CM (test group), both groups exhibited improved CAL, with no significant difference between them.^58^

 Studies evaluating gingival thickness (GT) demonstrated improvements across various techniques. The MCAT technique resulted in a significant increase in GT.^38,84^ When comparing CTG (control group) and CM (test group), both groups exhibited improved GT, with no significant difference between them.^58^

 Studies evaluating PD showed consistent stability across different techniques. Using a coronally advanced modified tunnel technique, no significant change in PD was reported.^34^ Similarly, the MCAT technique also resulted in PD remaining almost unchanged.^38^

 Studies evaluating gingival recession depth (GRD) and gingival recession width (GRW) demonstrated significant improvements across various techniques. The MCAT technique resulted in a significant reduction in both GRD and GRW.^28^ When comparing CTG (control group) and CM (test group), both groups exhibited improved GRD and GRW, with no significant difference between them.^58^

 Studies evaluating the plaque index (PI) and the gingival index (GI) have shown consistent stability. Using a coronally advanced modified tunnel technique, no significant changes in PI or GI were reported.^34^

####  Postoperative outcomes

 Postoperative outcomes were evaluated in several studies, with positive results reported across different techniques. Tambe et al^69^ achieved CRC and increased KTW with minimal postoperative complications using a modified TUN technique with a V-reverse suture. Also, Skierska et al^83^ demonstrated that adding cross-linked hyaluronic acid (HA) to the tunnel technique with CTG significantly improved outcomes compared to CTG alone. The HA-enhanced group demonstrated superior RC, a greater gain in KTW, and faster healing with reduced inflammation.

 According to Lin,^85^ the double-VISTA approach further optimized patient experiences, with higher satisfaction and lower postoperative pain. Similarly, Devkar et al^84^ demonstrated that the MCAT technique with DGG yielded predictable outcomes, including uneventful healing and enhanced aesthetic results. These findings align with outcomes from the standard VISTA technique,^94^ which incorporated a subperiosteal sling suture and horizontal mattress suture and improved the stability of the CTG and flap complex, leading to better RC and KTW.^50^

###  Coronally advanced flap for CTG stabilization 

####  Outcomes by clinical parameter

 The CAF technique, often combined with CTGs, has been widely studied for treating GR. Below, the findings are organized by clinical parameters and the studies that measured them.

 RC and CRC were evaluated across multiple studies using various suturing techniques. Studies using sling sutures reported improved RC,^20,25,32,33,44^with some also achieving improved CRC.^30,35^ Combining sling and interrupted sutures further enhanced RC,^21,23,73^ particularly in the CTG group, which showed higher CRC compared to other groups.^30,35^

 Studies using interrupted sutures alone have also demonstrated improved RC,^19,59^ while advanced techniques, such as continuous and vertical mattress sutures, have contributed to similaroutcomes.^57^ Additionally, the use of continuous vertical mattress and sling sutures resulted in improved RC,^24^ highlighting the effectiveness of advanced suturing methods.

 KTW outcomes varied across studies, depending on the suturing technique used. Studies employing sling sutures reported mixed results: Byun et al^32^ and Tal et al^20^ observed an increase in KTW, while Cardoso et al^60^ noted a decrease. For studies combining sling and interrupted sutures, Vilarrasa & Blasi^73^ and McGuire & Nunn^23^ reported an increase in KTW. Similarly, studies using interrupted sutures, such as those by Cordioli et al^19^ and Salem et al,^59^ also demonstrated an increase in KTW. Additionally, Chelearescu et al^57^ achieved an increased KTW with continuous and vertical mattress sutures, and Cheung & Griffin.^24^ reported improved KTW using continuous vertical mattress and sling sutures.

 RD and recession width (RW) significantly decreased across studies using various suturing techniques. Studies employing sling sutures reported reductions in RD and RW.^32,33,44,60^ Similarly, studies combining sling and interrupted sutures also observed reductions in RD.^21,73^ Additionally, the use of continuous vertical mattress and sling sutures resulted in reductions in RD.^24^

 CAL improved across studies using various suturing techniques. Studies employing sling sutures reported improvements in CAL.^25,32,33,44^ Similarly, studies combining sling and interrupted sutures also revealed improvements in CAL.^21^ Additionally, the use of continuous vertical mattress and sling sutures resulted in improvements in CAL.^24^

 GT and GTT were evaluated across studies using different suturing techniques. Studies employing sling sutures, such as that by Cardoso et al,^60^ reported an increase in GT. In contrast, those combining sling and interrupted sutures, including a study by Vilarrasa and Blasi,^73^ observed an increase in GTT. Similarly, studies using interrupted sutures, such as that by Salem et al,^59^ also demonstrated an increase in GTT.

 Regarding PD, studies using sling sutures, including those by Byun et al,^32^ Tal et al,^20^ and Zucchelli et al,^33^ reported no significant changes in PD. Similarly, studies combining sling and interrupted sutures, such as that by Carnio et al,^21^ also found no significant changes in PD. However, Cheung and Griffin^24^ observed a decrease in PD using continuous vertical mattress and sling sutures.

 PI and GI were evaluated in studies using sling sutures, with Byun et al^32^ reporting no significant changes in either PI or GI.

###  Other techniques

####  Outcomes by surgical technique

 Several studies have explored advanced flap techniques and alternative methods for RC, often combined with CTGs or other materials (Tables S2 and S3). Below, the findings are organized by surgical techniques and their associated outcomes.

 The TUN technique resulted in improved RC, increased GTT, and VD.^53^

 A novel technique combining the lateral pedicle with a tunnel flap achieved CRC and excellent esthetic outcomes for single deep recessions on mandibular incisors.^49^

 Comparing a semilunar coronally positioned flap with adhesive to CTG with micro-sutures, the CTG group showed significantly increased GTT, with no significant differences in RC between the groups. Both groups demonstrated improvements in RD, RW, KTW, PD, and CAL, though differences were not statistically significant.^31^

 The double papilla technique, using a sling and interrupted sutures, resulted in 100% RC.^55^

 Using the double pedicle technique with sling, cross sling, and interrupted sutures, significant improvements were observed in GR, CAL, KTW, and GTT, with 90% RC and 60% CRC.^22^ Another study using a similar technique achieved CRC rates of 50% (advanced recession), 67% (moderate recession), and 100% (slight recession).^16^

 PRF with CAF was used to treat Miller’s class I recessions, resulting in a significant increase in AG and 5 mm of CAL.^54^

 Comparing CAF with two releasing incisions to a modified technique using horizontal incisions, the CAF group showed significantly better RC (84.81% vs. 68.98% in the test group). Both groups demonstrated a significant reduction in GR, gain in CAL, and an increase in KTT, with no significant changes in other clinical parameters.^27^

 A new approach combining a modified tunnel technique with simultaneous frenuloplasty stabilized the CTG with internal mattress sutures and advanced the flap coronally using vertical double-crossed sutures, achieving CRC.^66^

 The lingually-tied horizontal mattress contouring suture, a new suturing technique, stabilized the CTG with a sling-like configuration, resulting in long-term graft survival and stabilization.^52^

 Two cases of combined regenerative and mucogingival treatment for deep intrabony defects used deproteinized bovine bone xenograft and CTG secured with horizontal mattress sutures, achieving remarkable RC, KTH, GTT, and CAL two years postoperatively.^47^

 Comparing CAF (using sling and interrupted sutures) to SCRF (left unbound without sutures), the CAF group showed better outcomes in CAL, RC, CRC, and esthetics, while the SCRF group demonstrated a significant increase in KTW.^40^

## Discussion

 The current scoping review aimed to investigate the impact of various suturing techniques on tissue stabilization and clinical healing outcomes following surgery. A total of 63 studies meeting the inclusion criteria were analyzed, with a focus on measuring KTW and GTT.

 Various suturing methods, including periosteal suture, interrupted suture, sling suture, mattress suture, cross-suture, and continuous suture, were examined to determine whether the technique used significantly influenced tissue stabilization.

 The information gathered in this study ranges from a collection of case report studies to RCTs where various parameters such as KTW, CAL, and PD have been investigated, with detailed information provided in the results section. Additionally, some studies have examined other parameters, such as tissue shrinkage and patient pain postoperatively. These parameters should be considered in future studies for further investigations.

 Key limitations included variability in suture materials, the Miller classification of GR, and surgical site selection in the dental area during surgery, as well as the duration of surgery across different studies, which could potentially reduce the study’s accuracy.

 Based on the provided search results, it appears that while some studies, such as that by Agusto et al,^49^ have mentioned the suture material, most studies do not specify the suture material or needle size used. According to Baghele^52^ the choice of suture depends on factors such as the biological interactions of the materials, tissue configuration, and the biomechanical properties of the wound. Carvalho et al^25^ used 5-0 polyglactin 910 Vicryl sutures for CTG stabilization using a sling suture technique. Baghele^52^ believe that, in various suturing techniques, 4-0, 5-0, or 6-0 absorbable sutures can be used. Furthermore, if the surgeon does not use magnifying instruments, 4-0 and 5-0 sutures are more comfortable to work with.

 One of the other challenges involves GR according to the Miller classification.

 Thirty-three articles focused on Miller class I GRs,^18-20,22-33,36,38,40,41,44,46,50,51,54,55,57,58,60-62,65,67,69^ twenty-nine worked on Miller class II GRs,^18-30,32,33,36,38,41,44,46,49,50,51,57,60-62,65,67^ and six determined class III GRs;^4,21,34,43,65,76,81^ Miller class IV recession was addressed in just one article.^76^ As we know, a higher Miller classification indicates more GR in that area, leading to lower expectations of CRC after surgery. Additionally, the surgical site is crucial because, for instance, performing surgery in the mandibular incisor region, due to poor mucogingival conditions of the lower jaw,^67^ especially on the lingual side, is significantly more challenging than surgery in other areas.

 Lastly, the duration of surgery is another influential factor. For instance, procedures using cyanoacrylate required less operating time due to its ease of application compared to traditional suturing techniques.^62,63^ Conversely, longer procedures were associated with sutures that required additional steps, such as sealing contact points with composite resin.^38^

 One of the discrepancies in this study was the lack of RCTs for some techniques, with only case reports available for certain methods. The gold standard for evaluating the effect of suturing techniques on tissue stability is RCTs in which all parameters are kept constant, and only the suturing techniques vary. This type of study design allows for a direct comparison of the efficacy of different suturing techniques while minimizing the influence of confounding factors. Based on our research, only one study has investigated the impact of various suturing techniques on grafts. However, this study only looked at the effect of suture type on the shrinkage of FGG and did not consider other factors.^56^

 Several factors, including the surgeon’s expertise and individual practices, significantly influence surgical outcomes in this context. Almeida et al^91^ mentioned that less experienced surgeons prefer to use “X” sutures anchored in the periosteum, while the modified technique eliminates the need for periosteal sutures.

## Conclusion

 The suturing technique did not appear to be a definitive factor in graft stabilization, which can be due to the existence of highly significant heterogeneity in the studies and other limitations mentioned. Hence, it is advisable to conduct additional controlled RCTs in this field to examine the impact of suture type on graft outcomes.

## Competing Interests

 The authors deny any conflicts of interest.

## Data Availability

 The data in this article are available upon request. You can contact the corresponding author to obtain the necessary data (z.afshar90@yahoo.com).

## Ethical Approval

 The protocol was registered in Open Science with the code 10.17605/OSF.IO/4YR9F. In addition, this study was approved by the Ethics Committee of Isfahan University of Medical Sciences with ethics code IR.MUI.DHMT.REC.1403.133.

## Supplementary Files


Supplementary file 1 contains Table S1-S3.
