# Supplementary material for: Efficacy of different suturing techniques on gingival grafts: A scoping review
Source: J Adv Periodontol Implant Dent. 2025 Sep 25;18(1):55–69. doi: 10.34172/japid.025.3805 (PMC12913211; doi:10.34172/japid.025.3805)
Supplement: Supplementary file 1 — contains Table S1-S3. [file japid-18-55-s001.pdf]

Table S1: Data extraction of papers using CTG for soft tissue modification based on type of flaps

<table>
<tr><th>Type of Flap</th><th>First Author (year)</th><th colspan="2">Type of Suture</th><th>Description</th></tr>
<tr><td rowspan="29">Tunnel Technique</td><td rowspan="2">Agrawal et al. (2021)[64]</td><td>CTG</td><td>NM</td><td rowspan="13"></td></tr>
<tr><td>Flap</td><td>Cross mattress + Sling</td></tr>
<tr><td rowspan="2">Cieślik-Wegemund et al. (2016)[46]</td><td>CTG</td><td rowspan="2">Sling</td></tr>
<tr><td>Flap</td></tr>
<tr><td rowspan="2">Danskin et al. (2023)[72]</td><td>CTG</td><td>NM</td></tr>
<tr><td>Flap</td><td>Sling</td></tr>
<tr><td rowspan="2">Dembowska & Drozdzik (2007)[26]</td><td>CTG</td><td>NM</td></tr>
<tr><td>Flap</td><td>Vertical mattress</td></tr>
<tr><td rowspan="2">Zuhr et al. (2021)[41]</td><td>CTG</td><td>NM</td></tr>
<tr><td>Flap</td><td>Double cross</td></tr>
<tr><td rowspan="2">Skierska et al. (2024)[83]</td><td>CTG</td><td rowspan="2">Sling</td></tr>
<tr><td>CTG+ HA</td></tr>
<tr><td rowspan="2">Salem et al. (2020)[59]</td><td>CTG</td><td>Interrupted</td></tr>
<tr><td>Flap</td><td>NM</td><td>Tunnel/ pouch</td></tr>
<tr><td rowspan="2">Aroca et al. (2010)[34]</td><td>CTG</td><td>NM</td><td rowspan="14">Modified tunnel technique</td></tr>
<tr><td>Flap</td><td>Horizontal mattress (suspended above the contact)</td></tr>
<tr><td rowspan="2">Lee et al. (2021)[65]</td><td>CTG + CM</td><td>interrupted</td></tr>
<tr><td>Flap</td><td>NM</td></tr>
<tr><td rowspan="2">Tambe et al. (2022)[69]</td><td>CTG</td><td>NM</td></tr>
<tr><td>Flap</td><td>V-reverse Sutured</td></tr>
<tr><td rowspan="2">Yaman et al. (2015)[43]</td><td>CTG</td><td>NM</td></tr>
<tr><td>Flap</td><td>Double cross over the contact</td></tr>
<tr><td rowspan="2">Aroca et al. (2013)[38]</td><td>CTG</td><td>Horizontal mattress suture</td></tr>
<tr><td>Flap</td><td>Sling above contact</td></tr>
<tr><td rowspan="2">Rakasevic et al. (2020)[58]</td><td>CTG</td><td>NM</td></tr>
<tr><td>Flap</td><td>Sling</td></tr>
<tr><td>Devkar et al. (2024)[84]</td><td>DGG</td><td>Sling suture</td></tr>
<tr><td>Do (2019)[50]</td><td>CTG</td><td>Subperiosteal Sling</td><td></td></tr>
</table>

		Flap	Horizontal mattress + Interrupted	Vestibular Incision Subperiosteal Tunnel Access (VISTA)
	Lin (2025)[85]	CTG	Sling Suture	double-VISTA (dual vestibular incisions and subperiosteal tunneling)
Coronally Advanced Flap (CAF)	Byun et al. (2009)[32]	CTG	Sling	
		Flap		
	Cardoso et al. (2021)[60]	CTG	NM	
		Flap	Sling	
	Damante et al. (2019)[51]	CTG	NM	
		Flap	Sling	
	Cheung & Griffin (2004)[24]	CTG	Sling	
		Flap	Continuous vertical mattress + Sling	
	Zucchelli et al. (2010)[33]	CTG	NM	
		Flap	Sling	
	Salem et al. (2020)[59]	CTG	NM	
		Flap	Interrupted	
	Uraz et al. (2015)[44]	CTG	NM	
		Flap	Sling or interrupted	
	Han et al. (2008)[28]	CTG	Interrupted	
		Flap	Interrupted + Sling	
	Khuntia et al. (2020)[54] (Case 2)	CTG	NM	
		Flap	Continuous Sling	
	Pini-Prato et al. (2010)[35]	CTG	NM	
		Flap	Sling or Interrupted	
	McGuire & Nunn (2003)[23]	CTG	Interrupted + Sling	
		Flap	NM	
	Chelarescu et al. (2020)[57]	CTG	Continuous	
		Flap	Vertical Mattress	
	Cortellini et al. (2009)[30]	CTG	Interrupted + compressing sling	
		Flap	Interrupted	
	Cordioli et al. (2001)[19]	CTG	Interrupted	
		Flap	Interrupted for releasing incisions	
	Carnio et al. (2002)[21]	CTG	Interrupted+ sling	
		Flap	Interrupted for releasing incisions	
	Rosetti et al.	CTG	NM	

	(2000)[18]	Flap	Interrupted	
	Cardaropoli et al. (2012)[36]	CTG	NM	
		Flap	Double loop sling	
	Tal et al. (2002)[20]	CTG	NM	Releasing incisions of the CAF secured with sling sutures.
		Flap	Sling	
	Carvalho et al. (2006)[25]	CTG	Sling	Modified Coronally Advanced Flap
		Flap	Sling	
	Vilarrasa & Blasi (2023)[73]	CTG	Interrupted + Periosteal Sling	Double Laterally CAF
		Flap	Interrupted + Sling +Horizontal mattress	
Lateral pedicle+ Tunnel (GPST)	Agusto et al. (2019)[49]	CTG	NM	
		Flap	Interrupted	
semilunar Coronally Positioned Flap	Bittencourt et al. (2009)[31]	CTG	NM	
		Flap	Microsuture	
Double Papilla	Bautista et al. (2022)[55]	CTG	NM	
		Flap	Sling + Interrupted	
Double Pedicle	Paolantonio (2002)[22]	CTG	Sling	
		Flap	Sling+ Interrupted+ Cross sling	
	Nelson (1987)[16]	CTG	NM	
		Flap	Sling	

CTG: Connective tissue graft; NM: Not mentioned.

Table S2: Data collection on allografts, xenografts, and auto-grafts except CTG.

Type of graft		Type of flap	First author (Year)	Type of suture		other points
Xenograft	Collagen Matrix (CM)	CAF	Cardaropoli et al. (2012)[36]	CM	NM	-
				Flap	Double loop sling + interrupted	
		Tunnel	Cieślik-Wegemund et al. (2012)[46]	CM	NM	-
				Flap	Sling	
			Aroca et al. (2013)[38]	CM	Horizontal mattress suture	Modified Coronally Advanced

				Flap	Sling above contact	Tunnel used
			Rakasevic et al. (2020)[58]	CM	interrupted	-
				Flap	Sling	
Autograft	PRF	CAF	Khuntia et al. (2020)[54] (Case 1)	PRF	NM	-
				Flap	Sling	
			Uraz et al. (2015)[44]	PRF	-	
				Flap	Sling or Interrupted	
	Platelet Concentrate graft (PCG)	CAF	Cheung & Griffin (2004)[24]	PCG	Sling	-
Allograft	Acellular Dermal Matrix (ADM)	CAF	Felipe et al. (2007)[27]	ADM	NM	For CAF with releasing used sling suture and interrupted suture in releasing incision. For CAF without releasing used interrupted sutures at papilla.
				Flap	Sling and interrupted	
			Tal et al. (2002)[20]	ADM	NM	Releasing incisions of the CAF secured with sling sutures.
				Flap	Sling	

CM: Collagen matrix; PRF: Platelet rich fibrin; PCG: Platelet concentrate graft; ADM: Acellular dermal matrix; CAF: Coronally advanced flap; NM: Not mentioned.

Table S3: Data extraction of papers using flap advancement without additional graft

Type of flap	First author (Year)	Type of suture	Description
Coronally Advanced Flap (CAF)	Khuntia et al. (2020)[54]	Sling	-
	Pini-Prato et al. (2010)[35]	Sling or Interrupted	-
	Cortellini et al. (2009)[30]	Sling with Interrupted	-
	Bittencourt et al. (2009)[31]	Without suture	Pressure with adhesive
	Moka et al. (2014)[40]	Sling with Interrupted	-
Tunnel	Rasperini et al. (2019)[53]	Sling	above the splinted contact point
Semilunar coronally repositioned flap	Moka et al. (2014)[40]	Without suture	-
